# Methylation Biomarker of Chronic Heavy Alcohol Consumption (HAC), but Not Acute HAC, Predicts All-Cause Mortality in Prostate, Lung, Colorectal and Ovarian Cancer Screening Trial

**DOI:** 10.3390/genes17010070

**Published:** 2026-01-06

**Authors:** Steven R. H. Beach, James A. Mills, Jeffrey D. Long, Robert A. Philibert

**Affiliations:** 1 Department of Psychology, University of Georgia, Athens, GA 30602, USA; srhbeach@uga.edu; 2 Department of Psychiatry, University of Iowa, Iowa City, IA 52242, USA; jim-mills@uiowa.edu (J.A.M.); jeffrey-long@uiowa.edu (J.D.L.); 3 Department of Biostatistics, University of Iowa, Iowa City, IA 52242, USA; 4 Behavioral Diagnostics, Coralville, IA 52241, USA

**Keywords:** cg05575921, DNA methylation, alcohol, mortality

## Abstract

Background: Due to variability in patterns of consumption as well as well-known difficulties in obtaining valid self-report from heavy drinkers, quantifying the effects of heavy alcohol consumption on mortality is challenging. Using a DNA methylation biomarker of chronic heavy alcohol consumption (HAC) named the alcohol T-score (ATS), we previously showed that chronic HAC was a strong predictor of mortality. However, whether there is a similar effect when measures of shorter-term heavy alcohol use, i.e., recent “binge” drinking, were used to predict mortality was not examined. This is a critical issue because most biomarkers of HAC assess only short-term HAC. Methods: Therefore, we examined the prediction of all-cause mortality from a DNA methylation biomarker of smoking (cg05575921), the ATS and a short-term biomarker of recent heavy alcohol use (cg07375256) in 708 subjects from the Prostate, Lung, Colorectal and Ovarian (PLCO) Cancer Screening Trial using Cox Proportional Hazards Regression Modeling. Models were compared using Akaike’s Information Criterion. Results: The ATS was the best single predictor among three-variable models that included controls for sex and age. Of the possible four variable models, the model consisting of age, sex, cg05575921 methylation and ATS best predicted mortality. The addition of cg07375256 methylation did not improve model performance. In sensitivity analyses using only participants who provided alcohol SR (*n* = 639), the importance of the ATS and cg05575921 was replicated. We also found that ATS values were higher among those who declined to provide self-report alcohol use, indicating that missing self-report data about alcohol intake are not missing at random, and sometimes reflects elevated alcohol consumption. Finally, cg05575921 methylation was strongly associated with ATS values but only weakly with alcohol SR and not at all with cg07375256 methylation. Conclusions: Accordingly, this study indicates a strong effect of chronic HAC, but not short-term HAC, on mortality, further highlights the limitations of self-reported alcohol use in the prediction of all-cause mortality and indicates the value of assessing HAC in addition to smoking.

## 1. Introduction

Quantifying the effects of alcohol consumption on health care outcomes is a challenge for both clinicians and researchers. Numerous studies have shown the unreliability of self-report in both clinical and research settings [1,2,3,4], with some variability among study populations [5]. Nonetheless, self-reported use remains a central method for quantifying HAC. The potential for unreliable self-report to have significant negative impact on public health policies has been evident for many years. Illustrating the problem, the United States government has two large-scale mechanisms for quantifying the consumption of alcohol. The first is self-reported (SR) alcohol consumption data collected by the Centers for Disease Control (CDC) via their Alcohol-Related Disease Impact (ARDI) program [6]. The second data collection mechanism reflects the “harder” data reflected in alcohol sales data collected by the Alcohol Epidemiologic Data System (AEDS) sponsored by the National Institutes of Alcohol Abuse and Alcoholism (NIAAA) [7]. Analyses of the relationship of the magnitude of self-report to actual alcohol sales over a twenty-year period of time by Nelson and colleagues found a 3.3-fold ratio of alcohol sales to reported consumption [8], suggesting a very large bias toward under-reporting alcohol consumption. Even allowing for unconsumed alcohol, it is clear that there is substantial under-reporting of consumption using SR. Since the CDC SR-based estimates are used as the basis of much of our understanding of the effect of alcohol on health, it seems clear that estimates of alcohol consumption’s impact on health outcomes could be affected [8]. Furthermore, to the extent that under-reporting is greater among those with the greatest consumption, this pattern of under-reporting could substantially skew predictive algorithms, leading to additional concerns about potentially unreliable risk estimates in many epidemiological studies.

Conceivably, estimates of the effects of alcohol consumption on key health outcomes in epidemiological studies could be improved through the use objective biomarkers of alcohol consumption. However, because the existing cadre of alcohol biomarkers differ in key performance characteristics including sensitivity, specificity and window of coverage, the choice of which assay is best to use will vary [9,10,11]. In particular, there may be differences in the patterns of association for indices that reflect recent heavy alcohol consumption (HAC) compared to those that may capture longer-term HAC. Assessments of alcohol use such as breathalyzer and serum alcohol levels can be very sensitive and can be used to quantitate recent consumption, but they are limited to detection of alcohol consumption in the past day. Ethyl glucuronide (EtG) determinations in blood or saliva can detect alcohol consumption up to 48 h after last intake [9], with limited information about quantity consumed. Assessments of phosphatidylethanol (PEth) have a nearly 100% sensitivity for detecting alcohol use in those admitted for alcohol detoxification [10]. But the window of detection is 4–10 days and these assays provide only limited information about the quantity of alcohol consumed [10]. Finally, serological assessments of carbohydrate-deficient transferrin (CDT) have a half-life of approximately 10 days and extend the window of alcohol consumption to 3 weeks, but are thought to be inferior to PEth in detecting moderate levels of recent alcohol consumption and are only semi-quantitative [9,12]. Accordingly, while countering potential self-report biases, most currently used non-self-report methods of alcohol consumption are best seen as measures of very recent or relatively recent HAC and have unknown association with longer-term HAC.

DNA methylation approaches offer an alternative method for quantitating alcohol consumption. Specifically, using methylation-sensitive digital polymerase chain reaction (MSdPCR), we have developed methods for detecting both recent and chronic heavy alcohol consumption (HAC) [11]. Recent HAC is associated with methylation at cg07375256 which reverts with a half-life of approximately 21 days [13]. Chronic HAC, i.e., the alcohol T-score (ATS), relies on DNA methylation at four CpG sites sensitive to alcohol consumption, each of which have methylation reversion half-lives of 3 months or greater [14].

In a recent comparison of CDT, cg07375256 and ATS markers to predict alcohol withdrawal seizures (AWS), we noted differences in prediction of AWS, as well as in association with smoking [13]. Specifically, the cg07375256 marker, which identified recent HAC, was better than both the CDT and ATS in predicting AWS, and both the CDT and the cg07375256 marker were much more weakly associated with cg05575921 than was ATS [13]. This led us to speculate that measures of recent HAC, i.e., CDT and cg07375256, may have different correlates and consequences than measures of chronic HAC, i.e., the ATS. If binge drinkers were less likely to smoke and identify as smokers, but more likely to have AWS, they might also have different healthcare needs and show different patterns of mortality risk. Unfortunately, the population studied in Andersen et al., 2023 were all admitted for alcohol detoxification and consideration of possible alcohol withdrawal [13]. As such, they were followed only short-term and the findings do not allow for direct examination of these issues.

In the current communication, we determined cg05575921, cg07375256 and ATS values in 708 subjects from the PLCO Cancer Screening Trial Study who served as controls for a recent examination of the relationship of smoking to lung cancer. This sample is ideal for examination of differential impact of different patterns of alcohol use because it is enriched for alcohol use relative to the general population. Of particular interest was whether recent HAC (cg07375256) and chronic HAC (ATS) had similar associations with excess mortality beyond the effect of smoking (cg05575921). If so, and if assessment of recent HAC predicted long-term mortality, then any of the several currently available non-self-report indices of alcohol consumption might enhance long-term mortality estimates relative to self-reported consumption. Conversely, if recent HAC (cg07375256) did not replicate the association of chronic HAC (ATS) with excess mortality, then most currently available non-self-report measures of alcohol consumption might also be expected to have limited utility in the prediction of excess mortality, to the extent that they are indices of recent HAC only. Also of interest was the association of recent HAC (cg07375256) with chronic HAC (ATS). For comparison with prior work we also examined the predictive value of self-reported HAC. We also examined whether those refusing to provide SR could be considered missing at random with regard to alcohol use patterns, using non-self-report indices of HAC to compare them with the rest of the sample.

## 2. Materials and Methods

The PLCO Cancer Screening Trial enrolled 154,877 subjects between the ages of 54 and 74 at ten sites across the United States between 1993 and 2004 [15]. After enrollment, subjects were phlebotomized to provide DNA for this and other studies, then followed until the subject death or until study closure in 2025. All subjects in the PLCO Cancer Screening Trial provided written informed consent. The overall project was approved by the Institutional Review Board at the National Cancer Institute and at each of the ten recruitment sites.

The clinical and biological data in this study are from participants who served as controls in a National Cancer Institute-sponsored study of the epigenetics of lung cancer that paired each lung cancer case with at least 1 μg of available DNA with up to 4 age-, sex-, race- and smoking history-matched controls (minimum number = 1, maximum = 4). The matching strategy was designed and implemented by the Etiologic and Early Marker Studies (EEMS) group of the NCI. The full delineation of subject selection process in the parent study that was carried out by EEMS is provided in the Supplementary Materials section (Supplemental Information) of the original report. Because of the high research value of DNA specimens from rare cancers such as ovarian, brain, gallbladder, fallopian tube, esophagus, bile duct, peritoneal, stomach, other biliary tract cancers, pancreas, small intestine, mesothelioma, multiple myeloma, liver, male breast cancer, head and neck cancers, these specimens were excluded from inclusion in the study. Detailed coded clinical data with respect to lung cancer outcomes, mortality, cause of mortality and related information were provided to the team by the EEMS statistical group.

Given the method of their selection, the participants are expected to have more smokers than a general population sample, and so also more chronic HAC. At the same time, those control subjects who died over the course of the study did not experience lung cancer or any of the excluded rare cancers.

After selection, 1 µg aliquots of selected DNA samples were plated into 96-well format by the Frederick Central Repository, then shipped to Behavioral Diagnostics (Coralville, IA, USA) for methylation determinations.

Substance Use Variables:

The average self-reported daily alcohol intake for each subject for the 12 months prior to study intake was taken from the PLCO Dietary Health Questionnaire (DHQ version 1.0, National Cancer Institute, 2007). This DHQ variable is a calculated variable that sums intake from all alcohol-containing beverages into a single variable. When appropriate, this intake is expressed as a standard daily drink, which is calculated by dividing the reported intake in grams per day (gm/day) by 14 gm, which is the amount of alcohol that is contained in a standard drink according to the Centers for Disease Control [16].

Assessment of cg05575921 methylation was conducted using methylation-sensitive digital polymerase chain reaction (MSdPCR) according to our standard procedure using digital PCR equipment from ThermoFisher (Hercules, CA, USA) [17,18]. In brief, DNA samples were bisulfite converted using a Epitect Fast 96 DNA Bisulfite (BS) Conversion kit (Qiagen, Germantown, MD, USA), then a 3 µL aliquot of the BS converted DNA was then pre-amplified, diluted 1:3000, and PCR amplified using fluorescent, dual labeled primer probe sets specific for cg05575921 (Behavioral Diagnostics, Coralville, IA, USA) in combination with digital PCR reagents and a QuantStudio Absolute Q Digital PCR System from ThermoFisher (Hercules, CA, USA). Then, methylation values ((C/C + T) ratios) were determined using the proprietary software, (Version 3.1) then exported to the study database.

Methylation determination at the four loci used to form the ATS (cg02583484, cg04987734, cg09935388 and cg04583842) and cg07375256 determinations were conducted using methylation-sensitive digital polymerase chain reaction (MSdPCR) according to a similar set of procedures to the above using digital PCR equipment from Bio-Rad (Hercules, CA, USA) and primer probes sets from Behavioral Diagnostics [13,19]. As for cg05575921, cg07375256 values were reported as a methylation percentage. In contrast, the ATS is the sum of z-scores of the four loci named above and is a zero-centered metric in abstinent populations [19].

Comparisons between groups of normally distributed continuous variables were conducted using *t*-tests, while groups of non-normally distributed continuous variables were compared using Wilcoxon testing [20]. Correlations between variables were measured using Pearson’s correlation co-efficient [20].

The primary analysis examined time to death from any cause using Cox proportional hazards modeling [21]. A series of models were fit for all participants (N = 708). Sensitivity analyses examined the just the subset of participants who provided information on their average daily alcohol consumption over the past 12 months (N = 639). For each series, the base model included only age and sex. Subsequent models identified which of the available predictors produced the best fitting three variable model, and then which four- and five-variable models best fit the data, examining whether change in Akaike’s information criterion (AIC) suggested meaningful improvement. The three non-self-report measures were examined first, and then all four substance use variables were examined (cg05575921, cg07375256, ATS and alcohol self-report). Model fit was assessed with Akaike’s information criterion (AIC) [22,23]. In general, differences of 2 or fewer AIC units are regarded as not being statistically meaningful [24].

In addition, the relative support for alternative models can shift when AIC differences are relatively small or when multiple models lie within the ∆AIC < 4–7 range. We therefore examined the sensitivity of model-selection conclusions to both sampling decisions, such as inclusion of those without SR, and minor model-specification changes, such as the inclusion/exclusion of correlated predictors [24].

## 3. Results

The relevant clinical and demographic characteristics of this subsampling of the PLCO study is shown in Table 1. The sample consists of 429 male and 279 female participants who served as controls for a study of the relationship of smoking to lung cancer. The subjects, who had an average age at baseline assessment in their mid-sixties, are largely White (87.1%), with significant representation of both Black (5.9%) and Asian (4.5%) Americans. Over the 25 years of follow-up, 286 (40.4%) of these subjects died.

Consistent with their serving as controls for a study of lung cancer, an elevated proportion of the sample 252 (35.6%) reported being current smokers. A total of 376 (53.1%) subjects reported being former smokers, while only 80 (11.3%) subjects denied ever being a regular smoker. A total of 69 (10%) of subjects declined to provide data on average alcohol intake. The data from those subjects who did provide alcohol self-report were highly skewed toward zero, with 70% of subjects (474 of 682) reported consuming one or fewer (15 gm/day) standard drinks per day and 18% reporting complete abstinence. Males reported significantly higher average daily alcohol consumption (16 gm ± 27 or 1.1 standard drinks, interquartile range (IQR) 0.8 to 19.7 gm/day) than did females (9 gm ± 31) or 0.6 standard drinks, IQR 0.3 to 6.7 gm/day Wilcoxon *p* < 0.0001).

**Figure 1:**
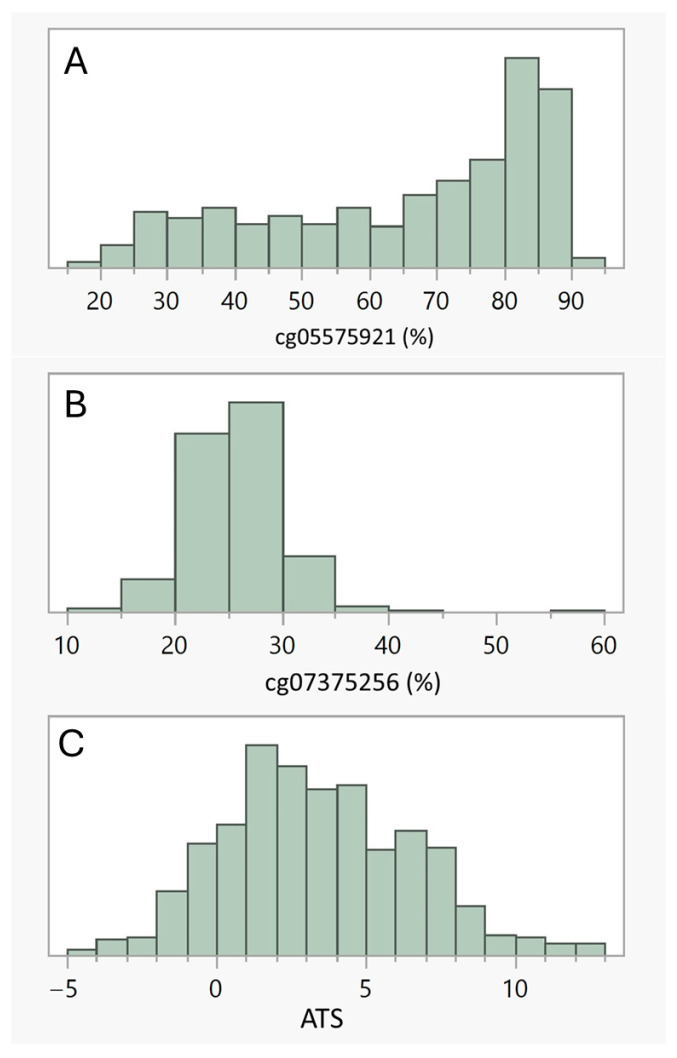
(**A**–**C**) illustrate the distribution of the three methylation indices, cg05575921, cg07375256 and ATS, respectively. Males had significantly lower cg05575921 methylation (64.0% ± 21.2), indicative of slightly more intense smoking histories, than females (68.3% ± 18.8, Wilcoxon *p* < 0.007). Cg07375256 values, which capture recent HAC, were significantly higher in males than in females (26.3 ± 4.0 vs. 24.6 ± 3.7, Wilcoxon *p* < 0.0001). Males also self-reported that they consumed more alcohol on a daily basis over the past 12 months than females (16 ± 27 gm/day vs. 9 ± 31 gm/day). However, there was only a trend for higher ATS values in males as compared to females (3.6 ± 3.2 vs. 3.1 ± 2.9, Wilcoxon *p* < 0.10).

Table 2 illustrates the linear correlations among the four substance use variables: cg05575921, ATS, cg07375256 and alcohol self-report while Figure 1 illustrates the distrbution of the methylation markers. The objective biomarker of smoking, cg05575921, was strongly correlated with the ATS, modestly correlated with alcohol SR over the past 12 months, but not correlated with cg07375256. Each of the alcohol indices were significantly related to one another with the strongest correlation (r = 0.18) being between the ATS and alcohol SR.

To assess the relationship of each of the methylation indices to all-cause mortality, we constructed a series of models using Cox proportional hazards modeling, then assessed their performance using AIC values. Table 3 shows the results from the analyses. The base model (Model 1), which consisted of sex and age, significantly predicted mortality. The addition of cg07375256 did not significantly improve model performance. However, the addition of cg05575921 and ATS values significantly improved model performance. As can be seen, the best fitting model with three predictors results from the addition of the ATS to sex and age (AIC = 3310). Simultaneous addition of both the ATS and recent HAC (cg07375256) did not significantly improve the model fit relative to ATS alone (Model 6; AIC = 3312). The addition of ATS and cg05575921 jointly to age and sex did result in an improvement over either added alone and resulted in the best fitting model using four predictors (Model 5; AIC = 3305; see Table 4 for the parameter estimates). Adding all predictors simultaneously (Model 7) did not result in an improved model (AIC = 3307).

The parameters for the best fitting model are given in Table 4. As the table indicates, each of the predictors made significant contributions to the final model.

As a sensitivity assessment, we next assessed the ability of self-report to improve prediction using the data from the 639 subjects for whom self-report data were available (See Table 5). In this subset of subjects, the basic finding with the full sample was replicated. The base model (Model 1) consisted of age and sex with the best fitting model including sex, age, ATS and cg05575921. The addition of recent HAC (cg07375256) or self-reported alcohol use did not further improve the model. However, as can be seen of Model 10 in Table 5, addition of self-reported HAC did not result in a poorer model fit relative to the best fitting four-variable model of sex, age, ATS and cg05575921.

We also assessed the relationship of providing self-report of alcohol use to the methylation metrics. Those not providing alcohol self-report information (*n* = 69) had significantly greater ATS (4.3 ± 3.4 vs. 3.3 ± 3.1, Wilcoxon *p* < 0.006) and lower cg05575921 (59.8% ± 21.9 vs. 66.3% ± 21.9, Wilcoxon *p* < 0.01) than those providing alcohol self-report data (*n* = 639), indicating greater chronic HAC and greater cigarette use. However, there was no difference in cg07375256 values between the two groups.

**Figure 2:**
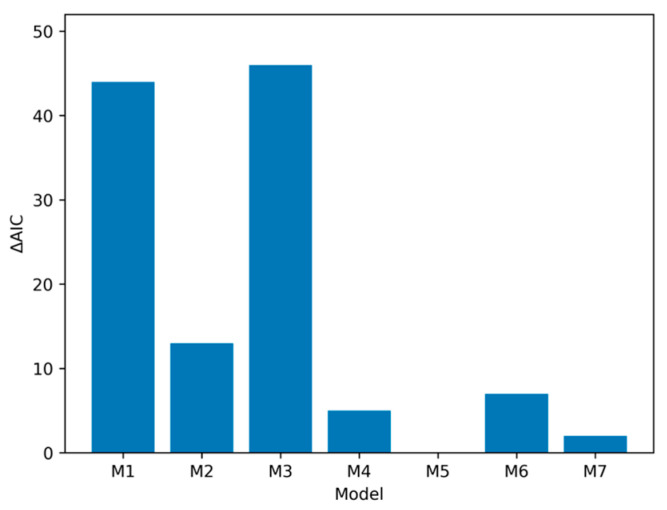
Comparison of ΔAIC-based changes in model performance. Model comparison in full sample (N = 708) shows that models including an index of chronic HAC (i.e., M4–M7) outperform models with sex, age, smoking and other alcohol indices. Note: ΔAIC for each model compared to M5. M1 (baseline) = Age + Sex; M2 = Age + Sex + cg05575921 (smoking); M3 =Age + Sex + cg07375256 (elevated short-term alcohol); M4 = Age + Sex + ATS (elevated long-term alcohol); M5 = Age + Sex + ATS + cg05575921 (Best fitting model); M6 = Age + Sex + ATS + cg07375256. M7 = Age + Sex + ATS + cg05575921 + cg07375256.

Finally, we graphed differences in Delta AIC across models to visualize differences in fit across model specifications. As can be seen in Figure 2, using model five as the best fitting model and comparing it to other model specifications, it can be seen that fit for model five is much better than the baseline model (Model 1) or the model using short-term HAC (Model 3), and is robustly better than the model using non-self-report of smoking (Model 2). However, it is less pronounced in its superiority to Models 4, 6 and 7, (i.e., all the other models including the ATS as a predictor).

**Figure 3:**
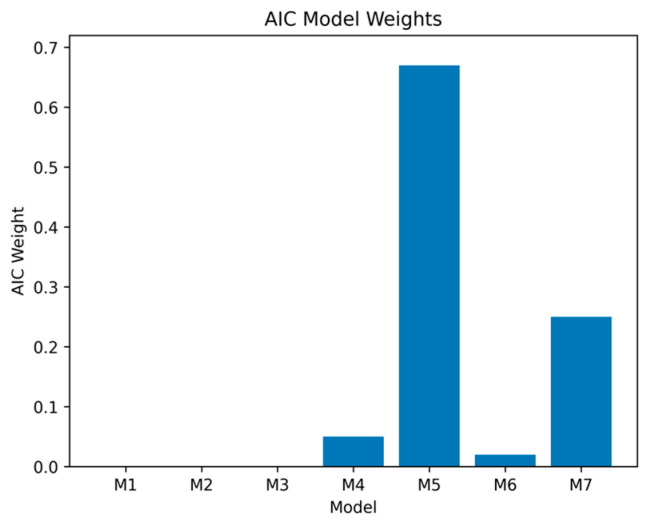
Modeling of AIC weights. The greatest probability (0.68) of being the best model to predict all-cause mortality in the full sample (N = 708) is Model 5. Note: Model weights were computed using the formula below—Akaike weight for model *i*.

To quantify the relative support for each candidate model, we also computed Akaike weights to provide a quantitative estimate of the probability that our best fitting model was likely to be robust in future replications. We transformed each model’s ΔAIC relative to Model 5 into a relative likelihood of being the correct model using the expression $exp[-\Delta_{i}/2]$, to capture the expected information loss associated with each model, and then normalized them so that they summed to 1, yielding model probabilities (equation provided in the text below Figure 3). As can be seen in Figure 3, the best fitting model that included both ATS and cg05575921 (i.e., Model 5) had an Akaike weight of 0.68, indicating a 68% probability of being the correct model. The next best model was the full five-variable model (Model 7), with a probability weight of 0.25; all remaining models had weights below 0.06, indicating minimal support. Evidence ratios further showed that Model 5 was approximately 2.7 times more likely to be correct than Model 7 and more than 12 times more likely than the best three-variable model (Model 4).

$$w_{i}=\frac{\exp\left(-\frac{1}{2}\Delta_{i}\right)}{\sum_{r=1}^{R}exp(-\frac{1}{2}\Delta_{r})}$$

where *R* is the number of competing models, Δ*_i_* is the AIC difference for model *i*, and *w_i_* is the Akaike weight for model *i*, interpreted as the relative probability that model *i* is the best model (in terms of Kullback–Leibler information) among the set considered.

Models M1 to M3 have negligible probabilities of being the correct model. M4 = Age + Sex + ATS (elevated long-term alcohol); M5 = Age + Sex + ATS + cg05575921 (Best fitting model), 68% likelihood of being the best model; M6 = Age + Sex + ATS + cg07375256. M7 = Age + Sex + ATS + cg05575921 + cg07375256.

## 4. Discussion

In summary, we show that chronic HAC, as captured by the ATS, but not short-term HAC captured by cg07375256 levels or alcohol self-report, predicted mortality over the subsequent 25 years among a subset of PLCO control participants. In this sample, cg05575921 methylation, a generally accepted measure of smoking intensity [25], was strongly inversely associated with the ATS, but not with cg07375256 methylation, indicating a strong positive relationship of smoking with long-term average alcohol intake, but not with short-term alcohol intake. Both chronic HAC (ATS) and smoking intensity (cg05575921 levels) made independent contributions to excess mortality. Strengths of this study include the use of a large, well described population sample, known to be at elevated risk for problematic health behaviors, especially smoking. Weaknesses include the cross-sectional nature of the DNA sampling, the exclusion of subjects who went on to develop lung cancer or rare forms of cancer, the lack of testing for proportionality of the Cox models and that the sample is not representative of the current general population.

Table-based comparisons and visual summaries of AIC weights and ΔAIC values (see Figure 2 and Figure 3) indicated clear differences in model support across the models examined. The baseline model containing only age and sex was outperformed by several of the extended models that included non-self-report indices of chronic HAC and smoking, with the strongest improvement observed when ATS was added to predictive models. Among the three-variable models, the (age, sex, ATS) specification provided the best fit to the data and was markedly better than the models including cg05575921 or cg07375256 alone as the third predictor. This pattern is consistent with prior work highlighting ATS as a robust biomarker capturing mortality risk (e.g., [26]).

The overall best fitting model in the full sample was a four-variable specification that included both ATS and cg05575921 (Table 3, Model 5; AIC = 3305). This model carried the largest Akaike weight (AICw = 0.68). The next most competitive specification was the full five-variable model including all predictors (Model 7; AIC = 3307; AICw = 0.25). All remaining models had weights below 0.06, providing minimal support. Evidence ratios indicated that Model 5 was approximately 2.7 times more likely than Model 7 to be the best model and more than 12 times more likely than the best three-variable model (Model 4). The relatively high weight for Model 7 suggests that cg07375256 may contribute some modest incremental information above ATS and cg05575921, though its influence is not decisive.

Although Model 5 had the highest degree of support, the ΔAIC difference between Models 5 and 7 (ΔAIC = 2) suggests that both models are statistically plausible. By contrast, ΔAIC values exceeding 5 for all other models indicate substantially diminished support and little likelihood they would be supported in future resamples. Importantly, the core structural conclusion was highly stable: the ATS emerged as the most informative predictor examined, beyond age and sex, across all plausible models.

In a prior examination of a smaller selection of these PLCO subjects, we established that the ATS predicted mortality [26]. However, because of the long-half lives of methylation reversion, the ATS is insensitive to recent HAC. This work not only further extends that analysis in a larger sampling but also allows us to, for the first time to the best of our knowledge, test simultaneously the effect of objective biomarkers of both long-term (ATS) and short-term (cg07375256) HAC on mortality. Of particular interest was whether these two patterns of drinking behavior would be distinguishable in their association with long-term excess mortality. These data, which show that long-term and short-term HAC have different patterns of associations with both smoking and mortality, suggest the strong likelihood that they are associated with different underlying biological changes as well as problematic health behaviors and psychosocial risk processes.

The data show that in these subjects, chronic HAC, but not short-term HAC, is significantly associated with elevated all-cause mortality. Since in prior studies we have shown a strong relationship between the ATS and coronary heart disease (CHD) and with CHD-related mortality [26], we would expect a relative increase in CHD-related deaths. Unfortunately, given the limited size and bias of the sample, it is difficult to make reliable estimates in the current sample. Hopefully, in future studies we will be able to gather a sizeable, random sampling of this or some other well-informed sample to provide those estimated.

Epigenetic studies have the potential to provide an objective understanding to desistance from HAC. Desistance is defined as the process of stopping or significantly reducing alcohol use [27]. It is important to note that because we do not have repeated measurement of chronic HAC and short-term HAC across multiple time points at the current time, we cannot say whether they show different patterns of desistance. At a minimum, however, there is likely to be some desistance of chronic HAC over a 25-year period in a sample of this age range. Similarly, because short-term HAC or “binge drinking” is also both sporadic and widespread [28], we would expect that if we were to gather additional time points from this sample, we would see that many of the subjects with currently unremarkable cg07375256 levels would show strong epigenetic evidence of recent binge drinking at some assessments and not others. If so, it is likely that the additional signal would increase the amount of variation in mortality explained by cg07375256. At the same time, however, it is important to note that “binge” drinking as defined by the National Institute on Alcohol Abuse and Alcoholism is much milder than the pattern identified by elevations of cg07375256. The NIAAA definition requires only “a pattern of drinking alcohol that brings blood alcohol concentration (BAC) to 0.08%—or 0.08 g of alcohol per deciliter—or higher.” For a typical adult, this pattern corresponds to consuming five or more drinks (male), or four or more drinks (female), in about two hours [29]. In contrast, the type of recent HAC detected by cg07375256, which has a half-life of reversion of approximately 21 days and was designed to predict alcohol withdrawal, is more sustained, with the pattern of heavy drinking lasting several days if not weeks [13]. Unfortunately, the frequency of that type of alcohol consumption in the general population is currently poorly understood. Given the nearly complete unreliability of ASR in this study, these data strongly suggest the need for epidemiologically sound examinations of the effects of alcohol use on mortality using a panel of biomarkers capable of capturing the range of alcohol consumption patterns.

Still, understanding these differences in patterns of desistance from the two forms of HAC identified by the two epigenetic tests could help more fully capture differential association with mortality. Because short-term HAC is, by definition, episodic, this one-time cross-sectional sampling may have captured only a fraction of the sample that could have been characterized as experiencing short-term HAC in their lifetimes. If so, this may also have suppressed our ability to detect the contribution of short-term HAC to mortality relative to a strategy using repeated assessments. This may be critically important given the role of “binge” drinking in accidental death [29,30]. Likewise, it may have underestimated the percentage of those with chronic HAC who would have eventually shown short-term HAC as well. In any case, these data strongly suggest that the use of epigenetics can bin or “lump” heavy drinkers into two groups: (1) those who are primarily short-term drinkers who tend not to smoke and (2) those who are chronic drinkers who as a group tend to smoke heavily. But this binning underplays the true complexity of substance use in this sample because despite the strong association of ATS and cg05575921, many subjects with high ATS levels have cg05575921 levels of 80% or greater, a level that is predictive of non-smoking status [31].

Interestingly, the data also suggest the intriguing possibility that smoking cessation may be a useful entry point to improve the health of those with chronic HAC. Our research and that of others have shown that when a subject stops smoking, they often stop drinking heavily [32,33,34]. There may be many reasons for this association, but if the impact of smoking cessation could be harnessed in the service of health promotion for those with chronic HAC, it suggests the potential for a substantial health impact with likely consequences for long-term mortality.

Since the impact of smoking and drinking on both personal health and public health expenditures is considerable, this makes a thorough understanding of the factors promoting dual use more imperative [35,36]. In that regard, cohorts such as the PLCO can make a vital contribution. Unfortunately, the biased nature of this PLCO subsample may also limit the conclusions that can be made for other purposes. Still, the larger sample had extensive initial characterization with many subjects participating in further longitudinal characterizations. As such, explorations of the factors that promote dual use may benefit from the use of this publicly available resource. Furthermore, these data strongly support the use of dual use cessation strategies and the need to test whether precision epigenetic guided financial incentive treatment (FIT) programs could reduce alcohol-related morbidity and mortality [34,37,38,39].

These data also add to an existing body of evidence suggesting that reliance on self-report of alcohol can result in misleading patterns of association in epidemiological surveys [1,3,5]. In part, as noted, this appears to reflect a strong tendency toward under-reporting alcohol use that attenuates associations. For example, Lakso and colleagues examined PEth levels in frozen blood samples (at −80 °C) in several large European epidemiological studies and found that on average, participants under-reported their alcohol consumption severalfold [37]. Since an ATS level of 2 is roughly equivalent to chronic intake of approximately two standard drinks per day, and the modal ATS value in this admittedly biased sampling of the PLCO is well above 3, this suggests that the subjects in the PLCO also significantly under-reported their consumption of alcohol severalfold. Also suggesting caution in reliance on self-report is our finding that the substantial number of subjects who declined to provide a self-report of alcohol use were also very elevated on the ATS scale as a group. This means that the most informative part of a sample regarding association of alcohol use and problematic outcomes is likely either under-reporting their use or declining to provide a report at all. It would not be surprising if this results in attenuated or non-significant associations of self-reported HAC with health and mortality outcomes.

In an ideal world, we would examine the correspondence between other non-self-report measures of HAC and the two methylation-based indices used in the current study. Regrettably, although there was enough DNA from these subjects (1 μg) to complete these studies, additional biological material for many of these subjects was not made available to us, limiting our ability to examine these associations. Our prediction for future efforts of this sort is that because they are more reflective of recent HAC than of chronic HAC, alternative non-self-report indices of HAC may have limited power to predict excess mortality. Nevertheless, given the strong impact of alcohol use on healthcare outcomes and the potential utility of each of different biomarkers for furthering our global understanding of alcohol use, we suggest that a simultaneous determination of multiple non-self-report biomarkers of HAC in a large cohort of well characterized subjects could lead to a firmer understanding of the clinical underpinnings and consequences of HAC. Similarly, closer examination of the similarities and differences in the metabolomic and proteomic consequences of recent vs. chronic HAC could also be useful in better understanding their different implications for mortality and morbidity.

## Figures and Tables

**Table 1:** Clinical and demographic characteristics of study sample.

	Male	Female
N	429	279
Age	63.0 ± 5.2	63.2 ± 5.3
Race		
White, non-Hispanic	365	252
Black, non-Hispanic	28	14
Hispanic	6	5
Asian	25	7
Pacific Islander	4	1
American Indian	1	-
SR Smoking Status		
Current	154	98
Former	238	138
Never	37	43
Died	202	84
SR Alcohol Consumption	16 ± 27	9 ± 31
(gms/day)		
0–50 gm	351	250
50–100 gm	26	2
>100 gm	8	2
No answer	44	25
Methylation Biomarkers		
cg05575921	64.0% ± 21.2	68.3% ± 18.8
cg07375256	26.3% ± 4.0	24.6% ± 3.7
ATS	3.6 ± 3.2	3.1 ± 2.9

**Table 2:** Pearson correlation coefficients of substance use variables.

	cg05575921	ATS	cg07375256	Alcohol SR
cg05575921	1	−0.61	−0.06	−0.15
ATS		1	0.08	0.18
cg07375256			1	0.08
Alcohol SR				1

SR = self-report, significant associations are bolded. Comparisons involving alcohol SR were limited to those 639 subjects for whom alcohol SR was available (*n* = 639).

**Table 3:** Proportional hazards model selection results for all 708 subjects.

Model		AIC	Model Rank
1	Age + Sex	3349	6
2	Age + Sex + cg05575921	3318	5
3	Age + Sex + cg07375256	3351	7
4	Age + Sex + ATS	3310	3
5	Age + Sex + ATS + cg05575921	3305	1
6	Age + Sex + ATS + cg07375256	3312	4
7	Age + Sex + ATS + cg05575921 + cg07375256	3307	2

SR = self-report, AIC = Akaike information criterion.

**Table 4:** Parameter estimates for proportional hazards model 5.

Variable	Parameter Estimate	Standard Error	*p*-Value
Age	0.120	0.013	<0.0001
Sex (M)	0.542	0.131	<0.0001
Cg05575921	−0.010	0.004	<0.008
ATS	0.092	0.023	<0.0001

M = male.

**Table 5:** Proportional hazards model selection results for 639 subjects with alcohol self-report (ASR).

Model		AIC	Model Rank
1	Age + Sex	2926	10
2	Age + Sex + cg05575921	2902	8
3	Age + Sex + cg07375256	2928	11
4	Age + Sex + ATS	2892	5 *
5	Age + Sex + ASR	2923	9
6	Age + Sex + ATS + cg05575921	2889	1 *
7	Age + Sex + ATS + cg07375256	2894	7
8	Age + Sex + ATS + ASR	2893	6
9	Age + Sex + ATS + cg05575921 + cg07375256	2891	3
10	Age + Sex + ATS + cg05575921 + ASR	2889	1 *
11	Age + Sex + ATS + cg05575921 + cg07375256 + ASR	2891	3

SR = self-report, AIC = Akaike information criterion, * Best for number of predictors.

## Data Availability

The methylation data for this are being deposited with the NCI CDAS database in accordance with NOT-OD-19-121 guidelines.

## Supplementary Materials

The following supporting information can be downloaded at: https://www.mdpi.com/article/10.3390/genes17010070/s1. Supplement One: EEMS study cohort selection process.

## Abbreviations

The following abbreviations are used in this manuscript:

PLCO	Prostate, Lung, Colorectal and Ovarian Cancer Screening Trial
HAC	Heavy alcohol consumption
ATS	Alcohol T-score
CDC	Centers for Disease Control
ARDI	Alcohol-related disease impact
MSdPCR	Methylation-sensitive digital polymerase chain
NIAAA	National Institutes of Alcohol Abuse and Alcoholism
NCI	National Cancer Institute
CDAS	Cancer Data Access System
EtG	Ethyl glucoronate
PEth	Phosphatidyl ethanolamine
CPD	Cigarettes per day
CDT	Carbohydrate-deficient transferrin
PY	Pack year
AIC	Akaike’s information criterion
SR	Self-report
ASR	Alcohol self-report
CpG	Cytosine-phospho-guanine
IQR	Interquartile range
