# Supplementary material for: Methylation Biomarker of Chronic Heavy Alcohol Consumption (HAC), but Not Acute HAC, Predicts All-Cause Mortality in Prostate, Lung, Colorectal and Ovarian Cancer Screening Trial
Source: Genes (Basel). 2026 Jan 6;17(1):70. doi: 10.3390/genes17010070 (PMC12840812; doi:10.3390/genes17010070)

Table S1: Labstudies Report of 2022-0014 Methylation to predict risk for lung cancer

Label	*Description*	*Exclusion/Matching*
**EEMS ID**	2022-0014	**Exclusions:**
**Short Name**	Methylation to predict risk for lung cancer	Intervention Arm
**Investigator**	Robert Philibert	Baseline Questionnaire
**Lead Programmer**	Chris Cunningham	Genetic Consent
**Site**	Lung	No Rare Cancers in Controls
**Outcome**	Lung Cancer	Follow Up
		DNA/Source Available
**Cases**	1538	
**Non-Cases**	4612	
**Case Definition**	Confirmed Cancer Prior to Censor Date	
**Control Definition**	Not Case	
**Population Overlap**	Not Applicable	
**Censor Date**	Not Applicable	
**Exit Date**	Lung Exit	
**Entry Date**	Randomization	
**Build Month**	nov22	
**Match Ratio**	3:1	
**Match Technique**	Individual matching only non-cases as controls.	
**Time on Study**	Days from Blood Draw to Dx/Exit	
**Additional Factors**	None	
**Match Comments**	None	
		**Matching Variables:**
**Analyte Category**	Chromosomal	Gender
**Analytes**	Methylation	Race
**Material Type**	T0 DNA	Age at Randomization (5 year intervals)
**Amount Requested**	1000 ng	Smoking History
**DNA Extraction Method**	Not Applicable	Fiscal Year of Randomization
**Analytic Lab**	Behavioral Diagnostics	
**Batching Scheme**	Not Applicable	
**QC Plan**	Not Applicable	
**Pop Status**	Pending	

Label	*Description*	*Exclusion/Matching*
**Replenishment Status**	Not Applicable	
**Batching Status**	Not Applicable	
**Results Status**	Pending	
**Results Disposition**	Not Complete	
**Binder Status**	Not Complete	
**Description**	Add methylation to previously derived data and develop a formula for predicting likelihood of developing Lung CA	

PLCO EEMS request 2022-0014 A Phase II extension of the use of cg05575921 Methylation to predict risk for lung cancer billed to DCP
saved as /prj/plcoims/labstudies/eems/lung/2022-0014.cg05575921.methylation/population/master/pop.nov22.d091423.sas
09/23/2023
Table S2: Exclusion table

	Case/Control Status		
	Non-Case	Case	Total
Exclusion Category			
1. All I-Arm Subjects	74764	2679	77443
2. Has BQ	73002	2608	75610
3. Has Genetic Consent	64929	2123	67052
4. No Rare Cancers	61558	2022	63580
5. Has T0 blood collection after trial entry	57048	1878	58926
6. Has Pre-DX T0 DNA/Source Available (Eligible)	47291	1538	48829
7. Matched at a 3 to 1 Ratio (Selected)	4612	1538	6150

PLCO EEMS request 2022-0014 A Phase II extension of the use of cg05575921 Methylation to predict risk for lung cancer billed to DCP
saved as /prj/plcoims/labstudies/eems/lung/2022-0014.cg05575921.methylation/population/master/pop.nov22.d091423.sas
09/23/2023
Table S3A: Demographics table

		All				Eligible				Selected			
		Case/Control Status				Case/Control Status				Case/Control Status			
		Non-Case		Case		Non-Case		Case		Non-Case		Case	
		N	%	N	%	N	%	N	%	N	%	N	%
Total		74764	100.00	2679	100.00	47291	100.00	1538	100.00	4612	100.00	1538	100.00
Exclusions	***Did the Participant Return the BQ?***												
	No	1762	2.36	71	2.65	0	0.00	0	0.00	0	0.00	0	0.00
	Yes	73002	97.64	2608	97.35	47291	100.00	1538	100.00	4612	100.00	1538	100.00
	Genetic Consent												
	Missing Data	1956	2.62	256	9.56	0	0.00	0	0.00	0	0.00	0	0.00
	None	6487	8.68	248	9.26	0	0.00	0	0.00	0	0.00	0	0.00
	Full Consent	20283	27.13	172	6.42	15858	33.53	128	8.32	1384	30.01	128	8.32
	Original Collection Only	44597	59.65	1972	73.61	31433	66.47	1410	91.68	3228	69.99	1410	91.68
	CDCC Buccal Collection Only	939	1.26	9	0.34	0	0.00	0	0.00	0	0.00	0	0.00
	Hawaii Post 4/1/2013	502	0.67	22	0.82	0	0.00	0	0.00	0	0.00	0	0.00
	Rare Cancer?												
	No	70704	94.57	2554	95.33	47291	100.00	1538	100.00	4612	100.00	1538	100.00
	Yes	4060	5.43	125	4.67	0	0.00	0	0.00	0	0.00	0	0.00
	Has 1ug Germline DNA												
	No	15267	20.42	741	27.66	403	0.85	19	1.24	21	0.46	19	1.24
	Yes	59497	79.58	1938	72.34	46888	99.15	1519	98.76	4591	99.54	1519	98.76
	Has 1ug Pre-DX T0 Germline DNA												
	No	23869	31.93	1426	53.23	419	0.89	425	27.63	21	0.46	425	27.63
	Yes	50895	68.07	1253	46.77	46872	99.11	1113	72.37	4591	99.54	1113	72.37
Matching	***Gender***												
	Female	38047	50.89	1056	39.42	24149	51.06	606	39.40	1818	39.42	606	39.40
	Male	36717	49.11	1623	60.58	23142	48.94	932	60.60	2794	60.58	932	60.60
	Race												
	White	64598	86.40	2274	84.88	42389	89.63	1362	88.56	4086	88.59	1362	88.56
	Black	3710	4.96	173	6.46	2263	4.79	99	6.44	297	6.44	99	6.44
	Other	6456	8.64	232	8.66	2639	5.58	77	5.01	229	4.97	77	5.01

		All				Eligible				Selected			
		Case/Control Status				Case/Control Status				Case/Control Status			
		Non-Case		Case		Non-Case		Case		Non-Case		Case	
		N	%	N	%	N	%	N	%	N	%	N	%
	Age At Randomization												
	<= 59	25232	33.75	617	23.03	17222	36.42	374	24.32	1124	24.37	374	24.32
	60-64	22947	30.69	836	31.21	14630	30.94	496	32.25	1502	32.57	496	32.25
	65-69	16665	22.29	789	29.45	9892	20.92	442	28.74	1311	28.43	442	28.74
	>= 70	9920	13.27	437	16.31	5547	11.73	226	14.69	675	14.64	226	14.69
	Cigarette Smoking Status												
	Ambiguous	1	0.00	.	.	0	0.00	.	.	0	0.00	.	.
	No Form	1762	2.36	71	2.65	0	0.00	0	0.00	0	0.00	0	0.00
	Not Answered	17	0.02	.	.	8	0.02	.	.	0	0.00	.	.
	Never Smoked Cigarettes	34747	46.48	246	9.18	23164	48.98	153	9.95	459	9.95	153	9.95
	Current Cigarette Smoker	7030	9.40	1045	39.01	4154	8.78	602	39.14	1804	39.12	602	39.14
	Former Cigarette Smoker	31207	41.74	1317	49.16	19965	42.22	783	50.91	2349	50.93	783	50.91
	Fiscal Year of Randomization												
	<= 1997	43114	57.67	1754	65.47	25134	53.15	905	58.84	2718	58.93	905	58.84
	1998+	31650	42.33	925	34.53	22157	46.85	633	41.16	1894	41.07	633	41.16
In Total GWAS Population													
No		14119	18.88	884	33.00	1164	2.46	160	10.40	137	2.97	160	10.40
Yes		60645	81.12	1795	67.00	46127	97.54	1378	89.60	4475	97.03	1378	89.60
Study Center													
University of Colorado		6388	8.54	195	7.28	4132	8.74	122	7.93	450	9.76	122	7.93
Georgetown University		3904	5.22	149	5.56	2329	4.92	80	5.20	245	5.31	80	5.20
Pacific Health Research and Education Institute (Honolulu)		5210	6.97	212	7.91	1381	2.92	42	2.73	123	2.67	42	2.73
Henry Ford Health System		11991	16.04	345	12.88	6718	14.21	177	11.51	608	13.18	177	11.51
University of Minnesota		13839	18.51	588	21.95	9141	19.33	351	22.82	1025	22.22	351	22.82
Washington University in St Louis		7201	9.63	316	11.80	4458	9.43	185	12.03	457	9.91	185	12.03
University of Pittsburgh		8114	10.85	352	13.14	5743	12.14	218	14.17	617	13.38	218	14.17
University of Utah		7042	9.42	136	5.08	5099	10.78	87	5.66	313	6.79	87	5.66
Marshfield Clinic Research Foundation		8070	10.79	297	11.09	5690	12.03	199	12.94	578	12.53	199	12.94
University of Alabama at Birmingham		3005	4.02	89	3.32	2600	5.50	77	5.01	196	4.25	77	5.01
Diabetes													
No Form		1762	2.36	71	2.65	0	0.00	0	0.00	0	0.00	0	0.00

	All				Eligible				Selected			
	Case/Control Status				Case/Control Status				Case/Control Status			
	Non-Case		Case		Non-Case		Case		Non-Case		Case	
	N	%	N	%	N	%	N	%	N	%	N	%
Not Answered	303	0.41	14	0.52	136	0.29	4	0.26	12	0.26	4	0.26
No	67053	89.69	2411	90.00	43776	92.57	1434	93.24	4293	93.08	1434	93.24
Yes	5646	7.55	183	6.83	3379	7.15	100	6.50	307	6.66	100	6.50
Race												
White, Non-Hispanic	64598	86.40	2274	84.88	42389	89.63	1362	88.56	4086	88.59	1362	88.56
Black, Non-Hispanic	3710	4.96	173	6.46	2263	4.79	99	6.44	297	6.44	99	6.44
Hispanic	1382	1.85	39	1.46	853	1.80	25	1.63	81	1.76	25	1.63
Asian	2701	3.61	90	3.36	1430	3.02	41	2.67	121	2.62	41	2.67
Pacific Islander	377	0.50	23	0.86	207	0.44	6	0.39	15	0.33	6	0.39
American Indian	197	0.26	8	0.30	128	0.27	5	0.33	11	0.24	5	0.33
Missing	1799	2.41	72	2.69	21	0.04	0	0.00	1	0.02	0	0.00
Fiscal Year Of Randomization												
1994	5454	7.29	236	8.81	2118	4.48	89	5.79	252	5.46	89	5.79
1995	11432	15.29	585	21.84	6291	13.30	275	17.88	806	17.48	275	17.88
1996	12914	17.27	481	17.95	7962	16.84	275	17.88	843	18.28	275	17.88
1997	13314	17.81	452	16.87	8763	18.53	266	17.30	817	17.71	266	17.30
1998	10359	13.86	325	12.13	6730	14.23	200	13.00	588	12.75	200	13.00
1999	11609	15.53	322	12.02	8025	16.97	235	15.28	725	15.72	235	15.28
2000	7852	10.50	235	8.77	6003	12.69	165	10.73	486	10.54	165	10.73
2001	1830	2.45	43	1.61	1399	2.96	33	2.15	95	2.06	33	2.15
# of Years Since Stopped Smoking Cigarettes												
No Form	1762	2.36	71	2.65	0	0.00	0	0.00	0	0.00	0	0.00
Not Answered	567	0.76	17	0.63	362	0.77	6	0.39	38	0.82	6	0.39
Not Applicable	34747	46.48	246	9.18	23164	48.98	153	9.95	459	9.95	153	9.95
0 Years	7030	9.40	1045	39.01	4154	8.78	602	39.14	1804	39.12	602	39.14
0 - <10 Years	6577	8.80	564	21.05	4068	8.60	318	20.68	443	9.61	318	20.68
10 - <20 Years	8045	10.76	377	14.07	5074	10.73	238	15.47	551	11.95	238	15.47
20 - <30 Years	7924	10.60	225	8.40	5148	10.89	145	9.43	587	12.73	145	9.43
30 - <40 Years	6327	8.46	110	4.11	4173	8.82	64	4.16	539	11.69	64	4.16
40+ Years	1785	2.39	24	0.90	1148	2.43	12	0.78	191	4.14	12	0.78
>= 25 pack years	54738	73.21	720	26.88	35046	74.11	396	25.75	2166	46.96	396	25.75

	All				Eligible				Selected			
	Case/Control Status				Case/Control Status				Case/Control Status			
	Non-Case		Case		Non-Case		Case		Non-Case		Case	
	N	%	N	%	N	%	N	%	N	%	N	%
<25 or missing												
>=25	20026	26.79	1959	73.12	12245	25.89	1142	74.25	2446	53.04	1142	74.25

PLCO EEMS request 2022-0014 A Phase II extension of the use of cg05575921 Methylation to predict risk for lung cancer billed to DCP
saved as /prj/plcoims/labstudies/eems/lung/2022-0014.cg05575921.methylation/population/master/pop.nov22.d091423.sas
09/23/2023
Table S3B: Cancer characteristics table

	All		*Selected*	
	Case/Control Status		*Case/Control Status*	
	Case		*Case*	
	N	*%*	*N*	*%*
Total	2679	100.00	1538	100.00
Confirmed Lung Cancer				
Confirmed cancer	2679	100.00	1538	100.00
Was Lung Cancer the First Diagnosed Cancer?				
No	431	16.09	212	13.78
Yes	2248	83.91	1326	86.22
Lung Cancer Type				
Non-Small Cell Lung Cancer	2348	87.64	1350	87.78
Small Cell Lung Cancer	331	12.36	188	12.22
Is This A Carcinoid Tumor?				
No	2679	100.00	1538	100.00
Lung Cancer Grade (ICD)				
Well differentiated; Grade I	418	15.60	250	16.25
Moderately differentiated; Grade II	428	15.98	243	15.80
Poorly differentiated; Grade III	727	27.14	398	25.88
Undifferentiated; Grade IV	151	5.64	77	5.01
T cell; T precursor	1	0.04	1	0.07
Unknown	954	35.61	569	37.00
Lung Cancer Metastasis				
No	1513	56.48	863	56.11
Yes	1124	41.96	654	42.52
Unknown	42	1.57	21	1.37
Lung Cancer Histopathologic Type				
Squamous Cell Carcinoma	554	20.68	297	19.31
Spindle Cell Carcinoma	6	0.22	2	0.13
Small Cell Carcinoma	317	11.83	180	11.70
Intermediate Cell Carcinoma	5	0.19	3	0.20

	All		Selected	
	Case/Control Status		Case/Control Status	
	Case		Case	
	N	%	N	%
Adenocarcinoma	932	34.79	557	36.22
Acinar Adenocarcinoma	28	1.05	19	1.24
Papillary Adenocarcinoma	17	0.63	13	0.85
Bronchioalveolar Adenocarcinoma	122	4.55	76	4.94
Adenocarcinoma w/Mucus Formation	39	1.46	20	1.30
Large Cell Carcinoma	91	3.40	49	3.19
Giant Cell Carcinoma	3	0.11	1	0.07
Clear Cell Carcinoma	1	0.04	0	0.00
Adenosquamous Carcinoma	26	0.97	16	1.04
Adenoid Cystic Carcinoma	1	0.04	1	0.07
Non-small cell (recoded)	43	1.61	31	2.02
Carcinoma NOS (recoded)	446	16.65	245	15.93
Mixed small and non-small cell (recoded)	3	0.11	1	0.07
Neuroendocrine NOS (recoded)	45	1.68	27	1.76
Lung Stage (AJCC 5th Edition)				
Missing data	142	5.30	82	5.33
Stage I	8	0.30	2	0.13
Stage IA	422	15.75	258	16.78
Stage IB	282	10.53	156	10.14
Stage II	1	0.04	1	0.07
Stage IIA	34	1.27	20	1.30
Stage IIB	124	4.63	74	4.81
Stage IIIA	253	9.44	134	8.71
Stage IIIB	261	9.74	150	9.75
Stage IV	819	30.57	472	30.69
Occult Carcinoma	2	0.07	1	0.07
Small cell	331	12.36	188	12.22
Lung Stage (AJCC 7th Edition)				
Missing data	141	5.26	81	5.27
Stage I	8	0.30	2	0.13
Stage IA	422	15.75	258	16.78

	All		Selected	
	Case/Control Status		Case/Control Status	
	Case		Case	
	N	%	N	%
Stage IB	279	10.41	155	10.08
Stage II	72	2.69	40	2.60
Stage IIA	37	1.38	21	1.37
Stage IIB	53	1.98	35	2.28
Stage III	17	0.63	10	0.65
Stage IIIA	331	12.36	184	11.96
Stage IIIB	167	6.23	91	5.92
Stage IV	819	30.57	472	30.69
Occult Carcinoma	2	0.07	1	0.07
Small cell	331	12.36	188	12.22

PLCO EEMS request 2022-0014 A Phase II extension of the use of cg05575921 Methylation to predict risk for lung cancer billed to DCP
saved as /prj/plcoims/labstudies/eems/lung/2022-0014.cg05575921.methylation/population/master/pop.nov22.d091423.sas
09/23/2023
Table S4A: DNA Availability based on line 5 - Cases

		Eligible									
		No		Yes							
		DNA Availability		DNA Availability							
		None		DESL Sufficient		Extracted DNA available		Buffy Coat available		All	
		N	%	N	%	N	%	N	%	N	%
All		340	100.00	774	100.00	78	100.00	686	100.00	1878	100.00
DESL confirmed?											
No		340	100.00	.	.	78	100.00	686	100.00	1104	58.79
Yes		.	.	774	100.00	.	.	.	.	774	41.21
Amount of DNA not at DESL (T0)	*Amount of DNA (EEMS)*										
0	*0*	131	38.53	40	5.17	.	.	180	26.24	351	18.69
	<1	1	0.29	.	.	.	.	3	0.44	4	0.21
	1+	201	59.12	625	80.75	.	.	503	73.32	1329	70.77
0	*0*	1	0.29	.	.	.	.	.	.	1	0.05
	1+	1	0.29	.	.	.	.	.	.	1	0.05
<1	*0*	3	0.88	.	.	.	.	.	.	3	0.16
1+	*0*	2	0.59	5	0.65	.	.	.	.	7	0.37
	1+	.	.	104	13.44	78	100.00	.	.	182	9.69
Buffy Coat Summary (T0)	*Any Buffy Coat Available (EEMS)*										
0	*No*	103	30.29	129	16.67	7	8.97	.	.	239	12.73
	Yes	144	42.35	545	70.41	16	20.51	.	.	705	37.54
>0	*No*	93	27.35	80	10.34	36	46.15	.	.	209	11.13
	Yes	.	.	20	2.58	19	24.36	686	100.00	725	38.60

PLCO EEMS request 2022-0014 A Phase II extension of the use of cg05575921 Methylation to predict risk for lung cancer billed to DCP
saved as /prj/plcoims/labstudies/eems/lung/2022-0014.cg05575921.methylation/population/master/pop.nov22.d091423.sas
09/23/2023
Table S4B: DNA Availability based on line 5 - Controls

		Eligible							
		No		Yes					
		DNA Availability		DNA Availability					
		None		Extracted DNA available		Buffy Coat available		All	
		N	%	N	%	N	%	N	%
All		9757	100.00	9000	100.00	38291	100.00	57048	100.00
Amount of DNA (T0)	*Amount of DNA (EEMS)*								
0	*0*	5022	51.47	.	.	21531	56.23	26553	46.55
	0	.	.	.	.	3	0.01	3	0.01
	<5	572	5.86	.	.	1532	4.00	2104	3.69
	5+	2889	29.61	.	.	14962	39.07	17851	31.29
0	*0*	22	0.23	.	.	.	.	22	0.04
	0	2	0.02	.	.	.	.	2	0.00
	5+	4	0.04	.	.	.	.	4	0.01
<5	*0*	107	1.10	.	.	.	.	107	0.19
	<5	297	3.04	.	.	208	0.54	505	0.89
	5+	725	7.43	.	.	55	0.14	780	1.37
5+	*0*	67	0.69	.	.	.	.	67	0.12
	<5	50	0.51	.	.	.	.	50	0.09
	5+	.	.	9000	100.00	.	.	9000	15.78
Buffy Coat Summary (T0)	*Any Buffy Coat Available (EEMS)*								
0	*No*	2171	22.25	722	8.02	.	.	2893	5.07
	Yes	5749	58.92	5871	65.23	.	.	11620	20.37
>0	*No*	1837	18.83	1587	17.63	.	.	3424	6.00
	Yes	.	.	820	9.11	38291	100.00	39111	68.56

PLCO EEMS request 2022-0014 A Phase II extension of the use of cg05575921 Methylation to predict risk for lung cancer billed to DCP
saved as /prj/plcoims/labstudies/eems/lung/2022-0014.cg05575921.methylation/population/master/pop.nov22.d091423.sas
09/23/2023
Table S4C: T0 Pre-DX Germline DNA Availability based on line 7 (Selected)

		Years from Randomization to DX/Exit																									**Total**
		0	*1*	*2*	*3*	*4*	*5*	*6*	*7*	*8*	*9*	*10*	*11*	*12*	*13*	*14*	*15*	*16*	*17*	*18*	*19*	*20*	*21*	*22*	*23*	*24*	
Case/Control Status	***Years from Draw to DX/Exit***																										
Non-Case	***2***	.	.	1	4	.	.	.	.	.	.	.	.	.	.	.	.	.	.	.	.	.	.	.	.	.	5
	3	.	.	.	15	1	.	.	.	.	.	.	.	.	.	.	.	.	.	.	.	.	.	.	.	.	16
	4	.	.	.	.	11	3	.	.	.	.	.	.	.	.	.	.	.	.	.	.	.	.	.	.	.	14
	5	.	.	.	.	.	17	2	.	.	.	.	.	.	.	.	.	.	.	.	.	.	.	.	.	.	19
	6	.	.	.	.	.	.	21	1	.	.	.	.	.	.	.	.	.	.	.	.	.	.	.	.	.	22
	7	.	.	.	.	.	.	.	37	4	.	.	.	.	.	.	.	.	.	.	.	.	.	.	.	.	41
	8	.	.	.	.	.	.	.	.	41	10	.	.	.	.	.	.	.	.	.	.	.	.	.	.	.	51
	9	.	.	.	.	.	.	.	.	.	69	13	.	.	.	.	.	.	.	.	.	.	.	.	.	.	82
	10	.	.	.	.	.	.	.	.	.	.	130	19	.	.	.	.	.	.	.	.	.	.	.	.	.	149
	11	.	.	.	.	.	.	.	.	.	.	.	158	26	.	.	.	.	.	.	.	.	.	.	.	.	184
	12	.	.	.	.	.	.	.	.	.	.	.	.	189	41	.	.	.	.	.	.	.	.	.	.	.	230
	13	.	.	.	.	.	.	.	.	.	.	.	.	.	271	40	.	.	.	.	.	.	.	.	.	.	311
	14	.	.	.	.	.	.	.	.	.	.	.	.	.	.	245	67	.	.	.	.	.	.	.	.	.	312
	15	.	.	.	.	.	.	.	.	.	.	.	.	.	.	.	188	81	.	.	.	.	.	.	.	.	269
	16	.	.	.	.	.	.	.	.	.	.	.	.	.	.	.	.	215	82	.	.	.	.	.	.	.	297
	17	.	.	.	.	.	.	.	.	.	.	.	.	.	.	.	.	.	422	85	.	.	.	.	.	.	507
	18	.	.	.	.	.	.	.	.	.	.	.	.	.	.	.	.	.	.	393	68	.	.	.	.	.	461
	19	.	.	.	.	.	.	.	.	.	.	.	.	.	.	.	.	.	.	.	271	57	.	.	.	.	328
	20	.	.	.	.	.	.	.	.	.	.	.	.	.	.	.	.	.	.	.	.	498	48	.	.	.	546
	21	.	.	.	.	.	.	.	.	.	.	.	.	.	.	.	.	.	.	.	.	.	369	27	.	.	396
	22	.	.	.	.	.	.	.	.	.	.	.	.	.	.	.	.	.	.	.	.	.	.	284	7	.	291
	23	.	.	.	.	.	.	.	.	.	.	.	.	.	.	.	.	.	.	.	.	.	.	.	78	2	80
	24	.	.	.	.	.	.	.	.	.	.	.	.	.	.	.	.	.	.	.	.	.	.	.	.	1	1
Case	***0***	78	6	.	.	.	.	.	.	.	.	.	.	.	.	.	.	.	.	.	.	.	.	.	.	.	84
	1	.	50	6	.	.	.	.	.	.	.	.	.	.	.	.	.	.	.	.	.	.	.	.	.	.	56
	2	.	.	60	13	.	.	.	.	.	.	.	.	.	.	.	.	.	.	.	.	.	.	.	.	.	73
	3	.	.	.	60	10	.	.	.	.	.	.	.	.	.	.	.	.	.	.	.	.	.	.	.	.	70
	4	.	.	.	.	51	10	.	.	.	.	.	.	.	.	.	.	.	.	.	.	.	.	.	.	.	61

		Years from Randomization to DX/Exit																									
		**0**	**1**	**2**	**3**	**4**	**5**	**6**	**7**	**8**	**9**	**10**	**11**	**12**	**13**	**14**	**15**	**16**	**17**	**18**	**19**	**20**	**21**	**22**	**23**	**24**	**Total**
	5	.	.	.	.	.	52	11	.	.	.	.	.	.	.	.	.	.	.	.	.	.	.	.	.	.	63
	6	.	.	.	.	.	.	97	6	.	.	.	.	.	.	.	.	.	.	.	.	.	.	.	.	.	103
	7	.	.	.	.	.	.	.	84	13	.	.	.	.	.	.	.	.	.	.	.	.	.	.	.	.	97
	8	.	.	.	.	.	.	.	.	91	10	.	.	.	.	.	.	.	.	.	.	.	.	.	.	.	101
	9	.	.	.	.	.	.	.	.	.	93	19	.	.	.	.	.	.	.	.	.	.	.	.	.	.	112
	10	.	.	.	.	.	.	.	.	.	.	88	6	.	.	.	.	.	.	.	.	.	.	.	.	.	94
	11	.	.	.	.	.	.	.	.	.	.	.	82	17	.	.	.	.	.	.	.	.	.	.	.	.	99
	12	.	.	.	.	.	.	.	.	.	.	.	.	73	8	.	.	.	.	.	.	.	.	.	.	.	81
	13	.	.	.	.	.	.	.	.	.	.	.	.	.	70	7	.	.	.	.	.	.	.	.	.	.	77
	14	.	.	.	.	.	.	.	.	.	.	.	.	.	.	62	10	.	.	.	.	.	.	.	.	.	72
	15	.	.	.	.	.	.	.	.	.	.	.	.	.	.	.	53	10	.	.	.	.	.	.	.	.	63
	16	.	.	.	.	.	.	.	.	.	.	.	.	.	.	.	.	49	5	.	.	.	.	.	.	.	54
	17	.	.	.	.	.	.	.	.	.	.	.	.	.	.	.	.	.	45	2	.	.	.	.	.	.	47
	18	.	.	.	.	.	.	.	.	.	.	.	.	.	.	.	.	.	.	41	2	.	.	.	.	.	43
	19	.	.	.	.	.	.	.	.	.	.	.	.	.	.	.	.	.	.	.	35	6	.	.	.	.	41
	20	.	.	.	.	.	.	.	.	.	.	.	.	.	.	.	.	.	.	.	.	28	1	.	.	.	29
	21	.	.	.	.	.	.	.	.	.	.	.	.	.	.	.	.	.	.	.	.	.	13	.	.	.	13
	22	.	.	.	.	.	.	.	.	.	.	.	.	.	.	.	.	.	.	.	.	.	.	5	.	.	5
Total		78	56	67	92	73	82	131	128	149	182	250	265	305	390	354	318	355	554	521	376	589	431	316	85	3	6150

PLCO EEMS request 2022-0014 A Phase II extension of the use of cg05575921 Methylation to predict risk for lung cancer billed to DCP
saved as /prj/plcoims/labstudies/eems/lung/2022-0014.cg05575921.methylation/population/master/pop.nov22.d091423.sas
09/23/2023
Table S4D: Buffy availability for protected DNA

		Overall Status 20Aug2023				All	
		Protected (<1.2ug)		Protected (<10ug)			
		N	%	N	%	N	%
All		15	100.00	34	100.00	49	100.00
Any Buffy Coat Available (EEMS)	*Buffy Coat Summary (T0)*						
No	*0*	.	.	14	41.18	14	28.57
	0.25	2	13.33	16	47.06	18	36.73
	0.5	4	26.67	.	.	4	8.16
Yes	*1*	9	60.00	4	11.76	13	26.53

PLCO EEMS request 2022-0014 A Phase II extension of the use of cg05575921 Methylation to predict risk for lung cancer billed to DCP
saved as /prj/plcoims/labstudies/eems/lung/2022-0014.cg05575921.methylation/population/master/pop.nov22.d091423.sas
09/23/2023
Table S5: Impact analysis based on line 7 (Selected)

					Case/Control Status		
					Non-Case	Case	Total
Total					4612	1538	6150
Dead?							
Not Confirmed Dead					2527	186	2713
Dead					2085	1352	3437
Rare Cancer?	1st Reported Cancer	2nd Reported Cancer	3rd Reported Cancer	Cancer type			
No	N/A	N/A	N/A	N/A	3554	.	3554
	Adrenal glands	N/A	N/A	N/A	1	.	1
	Bladder	N/A	N/A	N/A	79	.	79
		Female genital, other and unspecified	N/A	N/A	1	.	1
		Ill-defined sites	N/A	N/A	1	.	1
		Lung	N/A	N/A	.	20	20
			Breast	N/A	.	1	1
		Melanoma	N/A	N/A	1	.	1
		Prostate	N/A	N/A	11	.	11
			Lung	N/A	.	4	4
			Hematopoietic and reticulendothelial systems	N/A	1	.	1
			Ureter, urinary organs	N/A	2	.	2
		Connective, subcutaneous, and other soft tissues and peripheral nervous system	N/A	N/A	1	.	1
		Ureter, urinary organs	N/A	N/A	1	.	1
		Colorectum	Endometrium	Lung	.	1	1
		Don't know	N/A	N/A	1	.	1
		Not ascertained	N/A	N/A	2	.	2
			Kidney and renal pelvis	Ureter, urinary organs	1	.	1

					Case/Control Status		
					Non-Case	Case	Total
	Breast	N/A	N/A	N/A	125	.	125
		Bladder	N/A	N/A	1	.	1
			Lung	N/A	.	1	1
		Leukemia	N/A	N/A	1	.	1
		Lung	N/A	N/A	2	21	23
			Leukemia	N/A	.	1	1
		Melanoma	N/A	N/A	1	.	1
		Non-Hodgkin's lymphoma	Colorectum	Lung	1	.	1
		Colorectum	Lung	N/A	.	1	1
				Other	.	1	1
	Cervix	N/A	N/A	N/A	2	.	2
	Female genital, other and unspecified	N/A	N/A	N/A	2	.	2
	Hodgkins disease	N/A	N/A	N/A	1	.	1
		Lung	N/A	N/A	.	2	2
	Ill-defined sites	N/A	N/A	N/A	1	.	1
		Lung	N/A	N/A	.	1	1
	Kidney and renal pelvis	N/A	N/A	N/A	25	.	25
		Bladder	Lung	N/A	.	1	1
		Leukemia	N/A	N/A	1	.	1
		Lung	N/A	N/A	.	4	4
		Prostate	N/A	N/A	1	.	1
		Skin	N/A	N/A	1	.	1
		Hematopoietic and reticulendothelial systems	N/A	N/A	1	.	1
		Not ascertained	N/A	N/A	1	.	1
	Leukemia	N/A	N/A	N/A	32	.	32
		Breast	Lung	N/A	.	1	1
		Kidney and renal pelvis	Lung	N/A	.	1	1
		Lung	N/A	N/A	.	5	5
			Melanoma	N/A	.	1	1
		Melanoma	N/A	N/A	1	.	1

					Case/Control Status		
					Non-Case	Case	Total
Lung	N/A	N/A	N/A		36	1252	1288
	Bladder	N/A	N/A		1	4	5
		Prostate	N/A		.	1	1
	Bone	N/A	N/A		.	2	2
		Connective, subcutaneous, and other soft tissues and peripheral nervous system	N/A		.	1	1
	Breast	N/A	N/A		1	7	8
		Bladder	N/A		.	1	1
		Melanoma	N/A		.	1	1
	Hodgkins disease	N/A	N/A		.	1	1
	Ill-defined sites	N/A	N/A		.	1	1
	Kidney and renal pelvis	N/A	N/A		.	5	5
		Hematopoietic and reticulendothelial systems	Colorectum		.	1	1
	Leukemia	N/A	N/A		.	1	1
	Melanoma	N/A	N/A		1	6	7
	Non-Hodgkin's lymphoma	N/A	N/A		.	4	4
	Prostate	N/A	N/A		.	11	11
		Bladder	N/A		.	2	2
	Skin	N/A	N/A		.	3	3
	Thyroid	N/A	N/A		.	1	1
	Endometrium	N/A	N/A		.	1	1
	Hematopoietic and reticulendothelial systems	N/A	N/A		.	1	1
	Respiratory system	N/A	N/A		.	1	1
	Colorectum	N/A	N/A		.	8	8
		Bladder	N/A		.	1	1
	Other	N/A	N/A		.	1	1
	Not ascertained	N/A	N/A		1	7	8
Melanoma	N/A	N/A	N/A		73	.	73
	Bladder	N/A	N/A		1	.	1
		Lung	N/A		.	2	2

					Case/Control Status		
					Non-Case	Case	Total
		Breast	***N/A***	***N/A***	1	.	1
		Kidney and renal pelvis	***N/A***	***N/A***	1	.	1
		Leukemia	***N/A***	***N/A***	1	.	1
		Lung	***N/A***	***N/A***	.	15	15
			Hematopoietic and reticulendothelial systems	***N/A***	.	1	1
		Non-Hodgkin's lymphoma	***N/A***	***N/A***	1	.	1
		Prostate	***N/A***	***N/A***	2	.	2
			Bladder	***N/A***	1	.	1
			Kidney and renal pelvis	***Lung***	.	1	1
		Colorectum	***Bladder***	***N/A***	1	.	1
	Non-Hodgkin's lymphoma	***N/A***	***N/A***	***N/A***	36	.	36
		Hodgkins disease	***N/A***	***N/A***	1	.	1
		Kidney and renal pelvis	***N/A***	***N/A***	2	.	2
		Leukemia	***N/A***	***N/A***	1	.	1
		Lung	***N/A***	***N/A***	1	7	8
		Non-Hodgkin's lymphoma	***Lung***	***N/A***	.	1	1
		Prostate	***N/A***	***N/A***	1	.	1
			Lung	***N/A***	.	1	1
		Colorectum	***N/A***	***N/A***	1	.	1
			Lung	***N/A***	.	1	1
	Prostate	***N/A***	***N/A***	***N/A***	361	.	361
		Bladder	***N/A***	***N/A***	9	.	9
			Lung	***N/A***	.	1	1
			Non-Hodgkin's lymphoma	***Lung***	.	1	1
			Ureter, urinary organs	***N/A***	1	.	1
		Bone	***Colorectum***	***N/A***	1	.	1
		Kidney and renal pelvis	***N/A***	***N/A***	2	.	2
			Lung	***N/A***	.	1	1
		Leukemia	***N/A***	***N/A***	3	.	3
		Lung	***N/A***	***N/A***	2	83	85
			Non-Hodgkin's lymphoma	***N/A***	.	1	1

					Case/Control Status		
					Non-Case	Case	Total
			Other	N/A	.	1	1
		Melanoma	N/A	N/A	4	.	4
			Lung	N/A	.	1	1
		Non-Hodgkin's lymphoma	N/A	N/A	8	.	8
			Melanoma	N/A	1	.	1
		Skin	N/A	N/A	1	.	1
		Anus and anal canal	N/A	N/A	1	.	1
		Connective, subcutaneous, and other soft tissues and peripheral nervous system	N/A	N/A	1	.	1
		Hematopoietic and reticulendothelial systems	N/A	N/A	2	.	2
			Lung	N/A	.	1	1
		Ureter, urinary organs	N/A	N/A	1	.	1
		Colorectum	N/A	N/A	8	.	8
		Not ascertained	N/A	N/A	1	.	1
	Skin	N/A	N/A	N/A	2	.	2
		Leukemia	Non-Hodgkin's lymphoma	N/A	1	.	1
		Lung	N/A	N/A	.	1	1
	Thyroid	N/A	N/A	N/A	5	.	5
		Prostate	N/A	N/A	1	.	1
		Pelvis	N/A	N/A	1	.	1
		Not ascertained	Melanoma	N/A	1	.	1
	Uterus	N/A	N/A	N/A	2	.	2
	Vagina	N/A	N/A	N/A	1	.	1
		Lung	N/A	N/A	.	2	2
	Anus and anal canal	N/A	N/A	N/A	2	.	2
	Connective, subcutaneous, and other soft tissues and peripheral nervous system	N/A	N/A	N/A	5	.	5
		Lung	N/A	N/A	.	1	1
	Endocrine glands	N/A	N/A	N/A	1	.	1
	Endometrium	N/A	N/A	N/A	17	.	17
		Breast	N/A	N/A	1	.	1
		Lung	N/A	N/A	.	2	2

					Case/Control Status		
					Non-Case	Case	Total
		Non-Hodgkin's lymphoma	N/A	N/A	2	.	2
	Hematopoietic and reticulendothelial systems	N/A	N/A	N/A	17	.	17
		Bladder	Prostate	N/A	1	.	1
		Leukemia	N/A	N/A	4	.	4
		Lung	N/A	N/A	.	7	7
		Non-Hodgkin's lymphoma	N/A	N/A	1	.	1
		Colorectum	N/A	N/A	1	.	1
	Pelvis	Connective, subcutaneous, and other soft tissues and peripheral nervous system	N/A	N/A	1	.	1
		Not ascertained	N/A	N/A	1	.	1
	Penis	N/A	N/A	N/A	1	.	1
		Prostate	Lung	N/A	.	1	1
	Thymus	N/A	N/A	N/A	1	.	1
	Ureter, urinary organs	N/A	N/A	N/A	3	.	3
		Bladder	N/A	N/A	2	.	2
		Kidney and renal pelvis	N/A	N/A	1	.	1
		Melanoma	N/A	N/A	1	.	1
		Penis	Male genital, other and unspecified	N/A	1	.	1
	Colorectum	N/A	N/A	N/A	75	.	75
		Bladder	N/A	N/A	1	.	1
		Breast	N/A	N/A	3	.	3
		Kidney and renal pelvis	N/A	N/A	1	.	1
		Lung	N/A	N/A	.	10	10
			Not ascertained	N/A	.	1	1
		Melanoma	N/A	N/A	1	.	1
			Not ascertained	N/A	1	.	1
		Prostate	N/A	N/A	2	.	2
		Not ascertained	N/A	N/A	1	.	1
	Not ascertained	N/A	N/A	N/A	12	.	12
		Lung	N/A	N/A	3	2	5
		Prostate	N/A	N/A	1	.	1

					Case/Control Status		
					Non-Case	Case	Total
		Connective, subcutaneous, and other soft tissues and peripheral nervous system	***N/A***	***N/A***	2	.	2
		Respiratory system	***N/A***	***N/A***	1	.	1
		Colorectum	***N/A***	***N/A***	1	.	1

PLCO EEMS request 2022-0014 A Phase II extension of the use of cg05575921 Methylation to predict risk for lung cancer billed to DCP
saved as /prj/plcoims/labstudies/eems/lung/2022-0014.cg05575921.methylation/population/master/pop.nov22.d091423.sas
09/23/2023
Table S6A: Matching Report

						Selected			
						No	Yes		
						Case/Control Status	Case/Control Status		
						Non-Case	Non-Case	Case	Total
Total						1.05E6	4612	1538	1.05E6
Case Set	Gender	Race	Age at Rand	Smoking Status	Fiscal Year of Randomization				
1	Female	White	<= 59	Never Smoked Cigarettes	<= 1997	9310	18	6	9334
2	Female	White	<= 59	Never Smoked Cigarettes	1998+	18306	21	7	18334
3	Female	White	<= 59	Current Cigarette Smoker	<= 1997	9885	123	41	10049
4	Female	White	<= 59	Current Cigarette Smoker	1998+	16115	111	37	16263
5	Female	White	<= 59	Former Cigarette Smoker	<= 1997	22806	75	25	22906
6	Female	White	<= 59	Former Cigarette Smoker	1998+	49889	99	33	50021
7	Female	White	60-64	Never Smoked Cigarettes	<= 1997	27441	45	15	27501
8	Female	White	60-64	Never Smoked Cigarettes	1998+	12048	24	8	12080
9	Female	White	60-64	Current Cigarette Smoker	<= 1997	9697	126	42	9865
10	Female	White	60-64	Current Cigarette Smoker	1998+	4104	72	24	4200
11	Female	White	60-64	Former Cigarette Smoker	<= 1997	56753	156	52	56961
12	Female	White	60-64	Former Cigarette Smoker	1998+	24574	93	31	24698
13	Female	White	65-69	Never Smoked Cigarettes	<= 1997	12509	30	10	12549
14	Female	White	65-69	Never Smoked Cigarettes	1998+	14563	39	13	14615
15	Female	White	65-69	Current Cigarette Smoker	<= 1997	2778	81	27	2886
16	Female	White	65-69	Current Cigarette Smoker	1998+	1564	51	17	1632
17	Female	White	65-69	Former Cigarette Smoker	<= 1997	27282	132	44	27458
18	Female	White	65-69	Former Cigarette Smoker	1998+	20831	102	34	20967
19	Female	White	>= 70	Never Smoked Cigarettes	<= 1997	6762	27	9	6798
20	Female	White	>= 70	Never Smoked Cigarettes	1998+	6480	24	8	6512
21	Female	White	>= 70	Current Cigarette Smoker	<= 1997	330	33	11	374
22	Female	White	>= 70	Current Cigarette Smoker	1998+	506	33	11	550
23	Female	White	>= 70	Former Cigarette Smoker	<= 1997	7788	75	25	7888
24	Female	White	>= 70	Former Cigarette Smoker	1998+	8373	69	23	8465
25	Female	Black	<= 59	Current Cigarette Smoker	<= 1997	17	6	2	25

						Selected			
						No	Yes		
						Case/Control Status	Case/Control Status		
						Non-Case	Non-Case	Case	Total
26	Female	Black	<= 59	Current Cigarette Smoker	1998+	83	6	2	91
27	Female	Black	60-64	Never Smoked Cigarettes	<= 1997	9	3	1	13
28	Female	Black	60-64	Never Smoked Cigarettes	1998+	188	6	2	196
29	Female	Black	60-64	Current Cigarette Smoker	<= 1997	56	15	5	76
30	Female	Black	60-64	Current Cigarette Smoker	1998+	37	6	2	45
31	Female	Black	60-64	Former Cigarette Smoker	<= 1997	58	3	1	62
32	Female	Black	60-64	Former Cigarette Smoker	1998+	136	6	2	144
33	Female	Black	65-69	Current Cigarette Smoker	<= 1997	14	6	2	22
34	Female	Black	65-69	Current Cigarette Smoker	1998+	26	6	2	34
35	Female	Black	65-69	Former Cigarette Smoker	<= 1997	101	12	4	117
36	Female	Black	65-69	Former Cigarette Smoker	1998+	43	3	1	47
37	Female	Black	>= 70	Never Smoked Cigarettes	1998+	54	3	1	58
38	Female	Black	>= 70	Former Cigarette Smoker	<= 1997	14	3	1	18
39	Female	Black	>= 70	Former Cigarette Smoker	1998+	42	6	2	50
40	Female	Other	<= 59	Never Smoked Cigarettes	1998+	106	3	1	110
41	Female	Other	<= 59	Current Cigarette Smoker	<= 1997	13	3	1	17
42	Female	Other	<= 59	Current Cigarette Smoker	1998+	18	3	1	22
43	Female	Other	<= 59	Former Cigarette Smoker	1998+	166	6	2	174
44	Female	Other	60-64	Never Smoked Cigarettes	<= 1997	156	6	2	164
45	Female	Other	60-64	Never Smoked Cigarettes	1998+	68	3	1	72
46	Female	Other	60-64	Current Cigarette Smoker	<= 1997	3	3	1	7
47	Female	Other	60-64	Current Cigarette Smoker	1998+	14	9	3	26
48	Female	Other	60-64	Former Cigarette Smoker	<= 1997	132	9	3	144
49	Female	Other	65-69	Never Smoked Cigarettes	<= 1997	103	6	2	111
50	Female	Other	65-69	Never Smoked Cigarettes	1998+	59	3	1	63
51	Female	Other	65-69	Current Cigarette Smoker	<= 1997	4	6	2	12
52	Female	Other	65-69	Former Cigarette Smoker	<= 1997	32	3	1	36
53	Female	Other	>= 70	Former Cigarette Smoker	1998+	33	6	2	41
54	Male	White	<= 59	Never Smoked Cigarettes	<= 1997	4011	12	4	4027
55	Male	White	<= 59	Never Smoked Cigarettes	1998+	2421	6	2	2429
56	Male	White	<= 59	Current Cigarette Smoker	<= 1997	15875	141	47	16063

						Selected			
						No	Yes		
						Case/Control Status	Case/Control Status		
						Non-Case	Non-Case	Case	Total
57	***Male***	***White***	***<= 59***	***Current Cigarette Smoker***	***1998+***	20955	165	55	21175
58	***Male***	***White***	***<= 59***	***Former Cigarette Smoker***	***<= 1997***	52809	114	38	52961
59	***Male***	***White***	***<= 59***	***Former Cigarette Smoker***	***1998+***	59738	114	38	59890
60	***Male***	***White***	***60-64***	***Never Smoked Cigarettes***	***<= 1997***	16535	33	11	16579
61	***Male***	***White***	***60-64***	***Never Smoked Cigarettes***	***1998+***	1657	12	4	1673
62	***Male***	***White***	***60-64***	***Current Cigarette Smoker***	***<= 1997***	19389	210	70	19669
63	***Male***	***White***	***60-64***	***Current Cigarette Smoker***	***1998+***	6613	108	36	6757
64	***Male***	***White***	***60-64***	***Former Cigarette Smoker***	***<= 1997***	190131	297	99	190527
65	***Male***	***White***	***60-64***	***Former Cigarette Smoker***	***1998+***	39251	129	43	39423
66	***Male***	***White***	***65-69***	***Never Smoked Cigarettes***	***<= 1997***	10536	45	15	10596
67	***Male***	***White***	***65-69***	***Never Smoked Cigarettes***	***1998+***	1873	18	6	1897
68	***Male***	***White***	***65-69***	***Current Cigarette Smoker***	***<= 1997***	7582	141	47	7770
69	***Male***	***White***	***65-69***	***Current Cigarette Smoker***	***1998+***	2331	90	30	2451
70	***Male***	***White***	***65-69***	***Former Cigarette Smoker***	***<= 1997***	139163	315	105	139583
71	***Male***	***White***	***65-69***	***Former Cigarette Smoker***	***1998+***	28442	138	46	28626
72	***Male***	***White***	***>= 70***	***Never Smoked Cigarettes***	***<= 1997***	5794	27	9	5830
73	***Male***	***White***	***>= 70***	***Never Smoked Cigarettes***	***1998+***	1334	21	7	1362
74	***Male***	***White***	***>= 70***	***Current Cigarette Smoker***	***<= 1997***	510	33	11	554
75	***Male***	***White***	***>= 70***	***Current Cigarette Smoker***	***1998+***	440	45	15	500
76	***Male***	***White***	***>= 70***	***Former Cigarette Smoker***	***<= 1997***	36741	156	52	36949
77	***Male***	***White***	***>= 70***	***Former Cigarette Smoker***	***1998+***	5738	57	19	5814
78	***Male***	***Black***	***<= 59***	***Current Cigarette Smoker***	***<= 1997***	146	18	6	170
79	***Male***	***Black***	***<= 59***	***Current Cigarette Smoker***	***1998+***	356	21	7	384
80	***Male***	***Black***	***<= 59***	***Former Cigarette Smoker***	***<= 1997***	44	3	1	48
81	***Male***	***Black***	***<= 59***	***Former Cigarette Smoker***	***1998+***	426	12	4	442
82	***Male***	***Black***	***60-64***	***Never Smoked Cigarettes***	***1998+***	30	6	2	38
83	***Male***	***Black***	***60-64***	***Current Cigarette Smoker***	***<= 1997***	100	18	6	124
84	***Male***	***Black***	***60-64***	***Current Cigarette Smoker***	***1998+***	63	12	4	79
85	***Male***	***Black***	***60-64***	***Former Cigarette Smoker***	***<= 1997***	212	12	4	228
86	***Male***	***Black***	***60-64***	***Former Cigarette Smoker***	***1998+***	174	9	3	186
87	***Male***	***Black***	***65-69***	***Current Cigarette Smoker***	***<= 1997***	34	15	5	54

						Selected			
						No	Yes		
						Case/Control Status	Case/Control Status		
						Non-Case	Non-Case	Case	Total
88	Male	Black	65-69	Current Cigarette Smoker	1998+	35	18	6	59
89	Male	Black	65-69	Former Cigarette Smoker	<= 1997	134	12	4	150
90	Male	Black	65-69	Former Cigarette Smoker	1998+	248	18	6	272
91	Male	Black	>= 70	Current Cigarette Smoker	<= 1997	7	6	2	15
92	Male	Black	>= 70	Current Cigarette Smoker	1998+	5	6	2	13
93	Male	Black	>= 70	Former Cigarette Smoker	<= 1997	46	6	2	54
94	Male	Black	>= 70	Former Cigarette Smoker	1998+	118	15	5	138
95	Male	Other	<= 59	Current Cigarette Smoker	<= 1997	20	9	3	32
96	Male	Other	<= 59	Current Cigarette Smoker	1998+	119	9	3	131
97	Male	Other	<= 59	Former Cigarette Smoker	<= 1997	326	12	4	342
98	Male	Other	<= 59	Former Cigarette Smoker	1998+	696	12	4	712
99	Male	Other	60-64	Never Smoked Cigarettes	<= 1997	76	3	1	80
100	Male	Other	60-64	Current Cigarette Smoker	<= 1997	35	10	4	49
101	Male	Other	60-64	Current Cigarette Smoker	1998+	57	12	4	73
102	Male	Other	60-64	Former Cigarette Smoker	<= 1997	1034	27	9	1070
103	Male	Other	60-64	Former Cigarette Smoker	1998+	76	3	1	80
104	Male	Other	65-69	Never Smoked Cigarettes	1998+	103	9	3	115
105	Male	Other	65-69	Current Cigarette Smoker	<= 1997	19	12	4	35
106	Male	Other	65-69	Current Cigarette Smoker	1998+	8	3	1	12
107	Male	Other	65-69	Former Cigarette Smoker	<= 1997	161	9	3	173
108	Male	Other	65-69	Former Cigarette Smoker	1998+	47	3	1	51
109	Male	Other	>= 70	Never Smoked Cigarettes	<= 1997	23	3	1	27
110	Male	Other	>= 70	Never Smoked Cigarettes	1998+	19	3	1	23
111	Male	Other	>= 70	Current Cigarette Smoker	1998+	.	3	1	4
112	Male	Other	>= 70	Former Cigarette Smoker	<= 1997	82	9	3	94
113	Male	Other	>= 70	Former Cigarette Smoker	1998+	94	9	3	106

PLCO EEMS request 2022-0014 A Phase II extension of the use of cg05575921 Methylation to predict risk for lung cancer billed to DCP
saved as /prj/plcoims/labstudies/eems/lung/2022-0014.cg05575921.methylation/population/master/pop.nov22.d091423.sas
09/23/2023
Table S6B: Irregularly selected controls

caseset	Gender	Age At Randomization	Race	Cigarette Smoking Status	Fiscal Year of Randomization	Age At Randomization	Age At Randomization	Fiscal Year Of Randomization	Fiscal Year Of Randomization
33	Female	65-69	Black	Current Cigarette Smoker	<= 1997	66	64	1995	1995
47	Female	60-64	Other	Current Cigarette Smoker	1998+	61	59	2000	2000
51	Female	65-69	Other	Current Cigarette Smoker	<= 1997	69	67	1997	1998
51	Female	65-69	Other	Current Cigarette Smoker	<= 1997	69	68	1997	1999
69	Male	65-69	White	Current Cigarette Smoker	1998+	66	66	1999	1996
69	Male	65-69	White	Current Cigarette Smoker	1998+	66	65	1999	1997
75	Male	>= 70	White	Current Cigarette Smoker	1998+	71	71	2000	1997
75	Male	>= 70	White	Current Cigarette Smoker	1998+	71	68	1999	1999
75	Male	>= 70	White	Current Cigarette Smoker	1998+	70	69	1999	1998
75	Male	>= 70	White	Current Cigarette Smoker	1998+	70	69	1999	1999
75	Male	>= 70	White	Current Cigarette Smoker	1998+	70	68	1999	1999
75	Male	>= 70	White	Current Cigarette Smoker	1998+	71	71	1999	1997
75	Male	>= 70	White	Current Cigarette Smoker	1998+	71	71	1999	1997
82	Male	60-64	Black	Never Smoked Cigarettes	1998+	60	60	1998	1997
83	Male	60-64	Black	Current Cigarette Smoker	<= 1997	60	56	1997	1997
83	Male	60-64	Black	Current Cigarette Smoker	<= 1997	60	55	1997	1997
83	Male	60-64	Black	Current Cigarette Smoker	<= 1997	60	58	1997	1999
87	Male	65-69	Black	Current Cigarette Smoker	<= 1997	66	64	1996	1995
87	Male	65-69	Black	Current Cigarette Smoker	<= 1997	66	63	1996	1997
87	Male	65-69	Black	Current Cigarette Smoker	<= 1997	67	67	1997	1998
87	Male	65-69	Black	Current Cigarette Smoker	<= 1997	67	64	1997	1998
87	Male	65-69	Black	Current Cigarette Smoker	<= 1997	67	70	1997	1998
87	Male	65-69	Black	Current Cigarette Smoker	<= 1997	66	64	1996	1997
88	Male	65-69	Black	Current Cigarette Smoker	1998+	66	66	2000	1995
88	Male	65-69	Black	Current Cigarette Smoker	1998+	69	70	1999	1995
88	Male	65-69	Black	Current Cigarette Smoker	1998+	67	60	2000	1999
88	Male	65-69	Black	Current Cigarette Smoker	1998+	66	61	2000	2000
88	Male	65-69	Black	Current Cigarette Smoker	1998+	66	64	2000	2001
88	Male	65-69	Black	Current Cigarette Smoker	1998+	69	74	1999	1996
88	Male	65-69	Black	Current Cigarette Smoker	1998+	69	63	1999	1999
88	Male	65-69	Black	Current Cigarette Smoker	1998+	66	64	2000	2001

caseset	Gender	Age At Randomization	Race	Cigarette Smoking Status	Fiscal Year of Randomization	Age At Randomization	Age At Randomization	Fiscal Year Of Randomization	Fiscal Year Of Randomization
88	Male	65-69	Black	Current Cigarette Smoker	1998+	66	62	2000	2001
88	Male	65-69	Black	Current Cigarette Smoker	1998+	67	61	2000	2001
88	Male	65-69	Black	Current Cigarette Smoker	1998+	67	62	2000	2001
91	Male	>= 70	Black	Current Cigarette Smoker	<= 1997	72	63	1996	1995
91	Male	>= 70	Black	Current Cigarette Smoker	<= 1997	72	62	1996	1995
91	Male	>= 70	Black	Current Cigarette Smoker	<= 1997	72	62	1996	1996
92	Male	>= 70	Black	Current Cigarette Smoker	1998+	72	63	1998	1995
92	Male	>= 70	Black	Current Cigarette Smoker	1998+	72	61	1998	1998
92	Male	>= 70	Black	Current Cigarette Smoker	1998+	72	68	1998	1994
95	Male	<= 59	Other	Current Cigarette Smoker	<= 1997	57	74	1996	1995
95	Male	<= 59	Other	Current Cigarette Smoker	<= 1997	57	62	1996	1996
105	Male	65-69	Other	Current Cigarette Smoker	<= 1997	69	71	1996	1995
105	Male	65-69	Other	Current Cigarette Smoker	<= 1997	69	70	1996	1995
105	Male	65-69	Other	Current Cigarette Smoker	<= 1997	65	64	1995	1996
105	Male	65-69	Other	Current Cigarette Smoker	<= 1997	69	71	1996	1997

PLCO EEMS request 2022-0014 A Phase II extension of the use of cg05575921 Methylation to predict risk for lung cancer billed to DCP
saved as /prj/plcoims/labstudies/eems/lung/2022-0014.cg05575921.methylation/population/master/pop.nov22.d091423.sas
09/23/2023
Table S7: Case table

	All		*Selected*	
	Case/Control Status		*Case/Control Status*	
	Non-Case	*Case*	*Non-Case*	*Case*
Total	74764	2679	4612	1538
J_LUNG_CANCER				
No confirmed cancer	74764	.	4612	.
Confirmed cancer	.	2679	.	1538

PLCO EEMS request 2022-0014 A Phase II extension of the use of cg05575921 Methylation to predict risk for lung cancer billed to DCP

saved as /prj/plcoims/labstudies/eems/lung/2022-0014.cg05575921.methylation/population/master/pop.nov22.d091423.sas

09/23/2023

Figure S1 : Case Follow-up vs. Control Follow-Up (days)

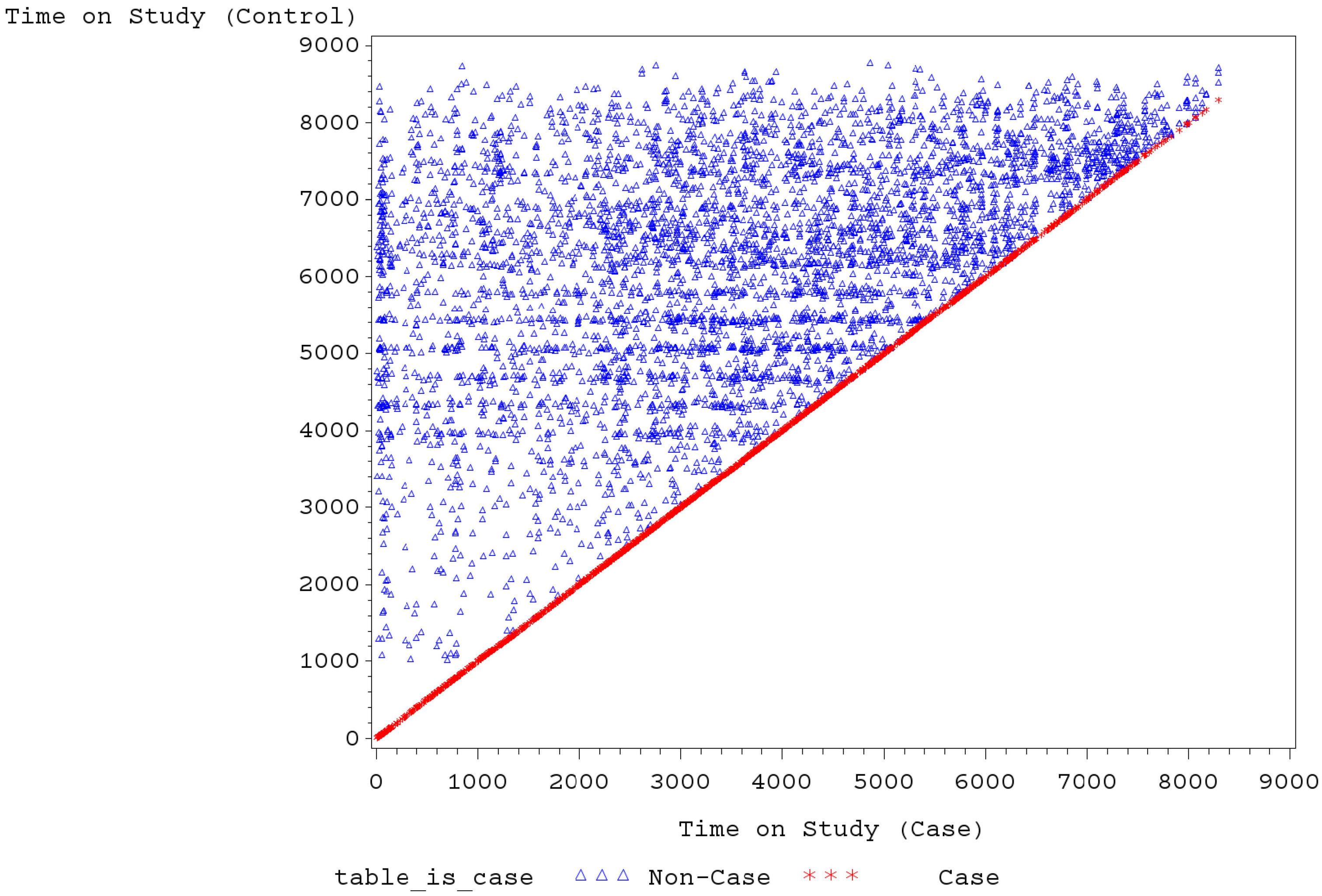

Supplement: Supplementary file 1 [file genes-17-00070-s001.zip › genes-4059668-supplementary.pdf]
